# Miscellaneous

**Published:** 1892-02

**Authors:** 


### MISCELLANEOUS.

### MOTHERS AS MATCH-MAKERS.

There is a kind of match-making which it is a mother's duty to attempt, writes Amelia E. Barr in the *Ladies' Home Journal.* But it has strict limitations. It resolves itself into the simple duty of introducing to her daughter young men whose moral character is good, who are in a position to marry, and who, physically, are not likely to repel her. The young people may then safely be left to their own instincts. There should be no attempt to coerce; no moral force used to make even a suitable marriage; though extremities may lawfully be used to prevent an evil marriage. A mother's match-making really begins while her daughter's education is in progress. And it is one of the strangest of facts that mothers generally force this education in the direction of those qualities likely to amuse young men—music, dancing, singing, dressing, playing games, chaffing wittily, etc. Now, such attractions are likely to procure plenty of flirtation; but young men rarely marry the girls they flirt with. And why do not mothers consider, most of all, that approaching period in their daughters' lives when they will, or ought to, cease being made love to? Why should the preparation for young ladyhood absorb all the girl's education? How many curriculums contain any arrangement for education for wifehood or parenthood? Yet, what man wishes to pass his life with a woman whose only charm is the power to amuse him? He might as wisely dine every day upon candy sugar.

### THE HUMAN HAIR.

We are constantly receiving enquiries as to the best means of preserving the hair, more recently from J. E. P., of Oswego, N. Y., and J. R. G., of Brownsville, Tenn. They come at all times and from all points far and near, from which we infer that *baldness* is not a *local* disease, except in individual instances, as in ours. Years ago, an observing physician warned us, on the shape of our head, that our top hair would take an early leave of us, and at this writing we are not called upon to brush away any stray locks that obstruct the line of vision. They have been brushed away a long time. It was only the other day that in having our hirsute border trimmed, not a word was said to us during the operation about some new discovery of an immaculate hair restorer, price $1 a bottle, just one left, etc., etc. Our surprise was immense. It was the only peaceful moment we had enjoyed for years, under such an operation, and we could not repress our astonishment. "Why! how is this?" we exclaimed. "You've skirmished round the timber line, where you could find any excuse for exercising your shears, and we hav'nt heard a word from you of some infallible lotion for bald pates. Have you become indifferent to the golden opportunities of your profession? or have you been brought under the benign influence of the Salvation Army, and ceased to prey on humankind?

We saw in the mirror before us that the Professor was in tears. A moment later he whipped off a superb wig and presented his smooth knob, as bright and shiny as a waning half-moon, sobbingly saying in broken accents, "I—I—tried it on my own head—it did bring out the hair, but never brought it back."

This then was the true solution of the diabolical mixture.

In all soberness the more common causes of baldness are insufficient exposure of the hair to the sun and air, close, ill-ventilated hats, excessive mental work and worry, the influence of hereditary alcoholic and other excesses, constant washing, and the neglect of the use of some proper stimulant at the roots. Children should, as much as possible, do without caps; and hats, when worn, should be roomy and of a light description. During the hot season, a stout hat is necessary for the prevention of sunstroke. A head-covering should never be worn indoors, in trains, or in closed carriages. The kind of material employed is of importance. In summer straw appears to be the best, on account of its lightness and permeability. In winter, hats made of light felt, well ventilated and unlined, are recommended. The ordinary tall and thick, heavy, unventilated hat cannot be too strongly condemned. Constant washing of the hair is unnecessary, as well as harmful. Once a week is quite often enough for cleanliness, as well as for maintaining the strength of the hair. The same remark applies to continual brushing, especially with hard brushes. There is a notion that greasing the hair is vulgar. After the hair has been washed, it is certainly beneficial to apply sparingly some form of simple grease or oil, otherwise it is apt to become dry and brittle. Bear in mind that every individual hair is a hollow tube whose life-essence is taken in at its roots by a purely natural process. Keep the scalp clean and moderately cool and let Nature have her way. A bald-headed Indian or cow-boy would be a curiosity.

Too frequent ablutions dry and deaden the hair by removing the natural oil. One washing a week is enough, and after the hair is dry it should be well brushed, but never with a very stiff brush, or one with metallic bristles. About once in two months the ends of the hairs should be clipped to prevent their splitting and to promote growth. Under no ordinary circumstances should a fine comb touch a child's head. Dandruff may be brushed out and washed out, but the fine comb, by irritating the scalp, produces the evil it is employed to remove. Even for the sake of beauty, hot curling irons should not be used for a child's hair. If it will not curl naturally, or with a little gentle coaxing, cut it short. It is said that rubbing a baby's hair the wrong way will make it curly. Whether this be true or not, the experiment can do no harm, even if it does no good.

### NEW YORK IN THE WORLD'S FAIR.

The MERCHANT'S COMMITTEE of the DRY GOODS TRADE of the CITY of NEW YORK, fully alive to the importance of their work, have issued a CIRCULAR LETTER to their brethren of the State, asking their co-operation to the end that the trade may be represented in a manner creditable to the Empire State. Amen to that.

NEW YORK'S GREAT FOOD EXPOSITION.—The citizens of New York propose to celebrate the discovery of America in their own way, assisted by representatives from every State and Territory in the Union. A great food show is to be held at Madison Square Garden in October of the present year. It is proposed at this Exposition to show the progress made by this country in the last four hundred years in our food supply. Ours is the greatest food-producing country in the world, and as food is the one thing above all others that first claims the attention of the human family, it is safe to predict that the coming Exposition will prove one of the most interesting and useful events of the century.

It will be the first affair of the kind ever held in the world. Only food products will be allowed on exhibition, exhibitors being restricted to manufacturers or producers, no dealer as such being allowed to participate. Every article of food exhibited must bear the bona fide name and address of its manufacturer, fictitious brands being rigidly excluded. Every manufacturer exhibiting must guarantee that his goods are the same as offered for sale to the public.

This is a move in the right direction.

CHEERFULNESS IN THE SICK-ROOM.—Despondency on the part of the patient is in many cases more deadly than disease, and whatever is said or done in and about the sick-room should be with a view to dispel that emotion, and replace it with something more healthful. Do not go tip-toeing and creeping about the apartment ; do not stand behind a screen, curtain, or door, and peer wistfully at the invalid ; do not stare fixedly at him from any point, and do not indulge in persistent questions which are evidently annoying. If the patient invites conversation, and is able to endure it, talk freely of those matters in which he is interested, the current news of the day, social events, or reminiscences ; but under no circumstances permit such topics as sickness, death, suffering, etc.

Matrimonial Club House.—A matrimonial club house is among recent innovations in a European city. It is a large roomy builing, divided into several apartments, in one of which portraits of each woman subscriber are exhibited, with description of her age, talents, fortune, color of hair, eyes, etc., size of hands and feet, and measurements of the bust, and general contour. There is also a brief account of her life.

In another room are the portraits of men candidates for connubial bliss.

A general reading room provides a medium for mutual meeting, and is presided over by an ancient dame who knits interminable stockings. There are also private rooms for more confidential tete-a-tetes.

One of the curious rules of the place is, that ladies only may enter the room where the men's portraits are, and men only are admitted to the women's gallery. They must meet in the common room. The establishment is conducted on moral principles, and the number of matches on its books approximate 1,000.

A Strange Custom.—A curious custom in connection with the disposal of the dead is practiced in the south of France. The people bury their dead in the ordinary course ; but after a lapse of years, when the bodies are reduced to dust, they dig up the bones, and, seemingly from a wish to economize ground, remove them from the grave in which they have hitherto rested, for another, and this time a final sepulchre. Piled up in a chamber near the church are numberless wooden coffers—some shaped like ordinary boxes, others fantastically carved, and some even fashioned to imitate shrines of churehes. In those boxes are curious openings in the form of hearts or clubs, giving passage to the air, though the contents have long since ceased to need a breathing hole. For in these boxes are kept the heads of those departed from this life. Generally each skull is in its own coffer, but sometimes two or three skulls of members of the same family are in the same chest. Upon each box is an inscription telling the curious whose head it is that lies within, and sometimes through the openings the grinning skull itself can be seen, a grey mass within its hard wooden shrine.

Bunions.—These painful enlargements and deformities of the feet should be treated, in the first instance, by warm fomentations or poultices, continued till the skin over the bunion is thoroughly softened ; a piece of lint saturated in extract of lead is then to be wrapped around the part, the lint being wetted with the extract as often as it dries. A square piece of thick buckskin, with a hole punched out of the middle, large enough to allow the bunion to pass through, and its surface spread with adhesive plaster, is then to be placed over the enlarged part, so that the pressure of the boot or shoe may be taken off the tender joint. By perseverance in this treatment when at home, and taking care that the plaster is thick enough to receive the pressure of the boot, a cure can always be effected. In old standing cases, after softening the skin by poultices, caustic should be rubbed over the calosity, and, after a few days, when the black cuticle peels off, re-applied till the excitement caused by the caustic induces gradual absorption of the indurated matter.

Strychnine to Cure Drunkenness.—A Russian physician named Portugaloff declares that strychnine is an infallible cure for drunkenness, administered in subcutaneous injections. He asserts that the experience of physicians has shown the cure to be as rapid as it is certain. The effect of the strychnine solution is to change the craving for drink into positive aversion, and this change is effected in a day. After a treatment of eight or ten days a patient may be discharged. The strychnine is administered by dissolving one grain in 200 drops of water, and injecting five drops of the solution every twenty-four hours.

